# The ring plus project: safety and acceptability of vaginal rings that protect women from unintended pregnancy

**DOI:** 10.1186/s12889-015-1680-y

**Published:** 2015-04-10

**Authors:** Céline Schurmans, Irith De Baetselier, Evelyne Kestelyn, Vicky Jespers, Thérèse Delvaux, Stephen K Agaba, Harry van Loen, Joris Menten, Janneke van de Wijgert, Tania Crucitti, for the RING PLUS study group

**Affiliations:** Institute of Tropical Medicine, Nationalestraat 155, 2000 Antwerp, Belgium; Rinda Ubuzima, KN 50th Av., Kiyovu, Kigali, Rwanda; Institute of Infection and Global Health, University of Liverpool, Ronald Ross Building, 8 West Derby Street, Liverpool, UK

**Keywords:** Contraceptive vaginal rings, Multi-purpose, Microbiome, Biofilm, Safety, Acceptability, Research capacity

## Abstract

**Background:**

Research is ongoing to develop multipurpose vaginal rings to be used continuously for contraception and to prevent Human Immunodeficiency Virus (HIV) infection. Contraceptive vaginal rings (CVRs) are available in a number of countries and are most of the time used intermittently i.e. three weeks out of a 4-week cycle. Efficacy trials with a dapivirine-containing vaginal ring for HIV prevention are ongoing and plans to develop multi-purpose vaginal rings for prevention of both HIV and pregnancy have been elaborated. In contrast with the CVRs, multi-purpose vaginal rings will have to be used continuously. Women who continuously use a CVR will no longer have menses. Furthermore, some safety aspects of CVR use have never been studied in-depth in the past, such as the impact of the vaginal ring on the vaginal microbiota, biofilm formation and induction of inflammation. We studied acceptability and these novel aspects of safety in Rwandan women. Although significant progress has been made over the past decade, Rwanda still has a high unmet need for contraception (with 47% unplanned births) and a generalized HIV epidemic, and CVRs are not yet available.

**Methods:**

We will conduct an open label, single centre, randomized controlled trial. A total of 120 HIV-negative women will be randomized to intermittent CVR use (to allow menstruation) or continuous CVR use. Women will be followed for a maximum of 14 weeks. In parallel, we will conduct a qualitative study using in-depth interview and focus group discussion methodology.

**Discussion:**

In addition to evaluating the safety and acceptability of intermittent and continuous CVR use in Rwandan women, we hope that our findings will inform the development of future multipurpose vaginal rings, will prepare Rwandan study populations for future clinical trials of multipurpose vaginal rings, and will pave the way for introduction of CVRs on African markets.

**Trial registration:**

Clinicaltrials.gov NCT01796613. Registered 14 February 2013.

## Background

Vaginal rings are polymeric drug delivery devices designed to provide controlled release of drugs for intravaginal administration over extended periods of time. Compared to systemic dosing, this maximizes efficacy at lower doses, thereby reducing side effects [1-8]. Furthermore, the ring can stay in place for 1–3 months, which improves adherence [9-11]. In recent years, vaginal rings have become popular for contraception and estrogen replacement therapy in Latin America, the United States and Europe where studies on the acceptability of the contraceptive vaginal ring (CVR) have shown overall good adherence and acceptability. On average, 85% of the users were satisfied or very satisfied and 85% to 90% would recommend the method to others [3,12].

However, CVRs are not yet on the market in any African country. Limited vaginal ring acceptability data are available in the context of HIV prevention research. A study by the International Partnership for Microbicides (IPM) in Tanzania and South Africa evaluated the acceptability of a vaginal ring without any active drugs [13]. The study found that the vast majority of women felt comfortable inserting and removing the ring, acceptability increased with prolonged use, and only one male sexual partner felt the ring during sex [14]. This placebo ring and a similar ring containing the antiretroviral drug dapivirine are currently used in two HIV prevention efficacy trials in African women (the IPM027 [15] and MTN020 [16] trials). CONRAD is currently developing a contraceptive/microbicide CVR (similar to NuvaRing®) and reformulating tenofovir gel with contraceptive properties for use with the SILCS diaphragm [17]. Given these developments, we believe that single-purpose and/or multi-purpose vaginal rings will eventually be introduced in African settings.

A multi-purpose vaginal ring will have to be used continuously, which is different from the way CVRs currently are mostly used. Current use is intermittent, i.e. the ring is taken out for one week after having been in place for three weeks. Women who continuously use a CVR will no longer have menses. We need in-depth data on the acceptability of the vaginal ring and of continuous use. Gaining a better understanding of acceptability requires the assessment of an intertwined set of variables and constructs such as users’ personal characteristics and factors influencing the user experience (product efficacy, attributes, and price; relationship context and factors impacting on sexuality). These factors in turn are embedded in a specific sociocultural context. The conceptual framework developed by Woodsong et al. for assessing the vaginal ring for microbicide delivery will guide our acceptability research [18].

Bacterial vaginosis (BV) is an imbalance of the vaginal microbiome. Epidemiological studies have shown that women with BV are twice more likely to acquire HIV than women without BV [19,20]. The vaginal microbiome can now be characterized in much more detail using molecular methods than was previously possible using microscopy. Very recent research has shown that the vaginal microbiome in African women can be categorized into at least 6 different bacterial communities, each with distinct characteristics in terms of the types and quantities of bacteria present, whether or not inflammation is induced and whether there is an increased risk for Human Immunodeficiency Virus (HIV) or other Sexually Transmitted Infections (STIs) [21]. The presence of inflammation is particularly important because inflammation often causes symptoms (itching, burning) and is associated with an influx of HIV target cells e.g. CD4+ T cells and macrophages into the genital area [22]. It is therefore very important that any product used inside the vagina (such as a CVR) does not cause irritation and/or inflammation.

BV has also been associated with biofilm formation of microorganisms on the vaginal epithelia and this may be why BV recurrence rates are very high after initial successful treatment [23,24]. BV is more common in African and African-American women, which could be due to the high prevalence of vaginal anaerobic bacteria that are prone to form biofilms [25,26]. It is also well known that biofilms easily form on the surface of catheters and prostheses [27]. Knowledge is lacking on whether vaginal bacteria can adhere to the ring and form a biofilm. A small study among four non-human primates suggested that the vaginal ring could lead to biofilm formation [28]. We therefore need to explore the formation of biofilms on vaginal rings in the vagina for different periods of time. Furthermore, biofilms may hamper delivery of the active compounds of the rings and could lead to clinical BV (which increases risk of HIV acquisition). Better knowledge on biofilm formation in the cervicovaginal context will aid further development of multi-purpose vaginal rings.

In Africa, especially in Rwanda there is an unmet need for contraception with 47% unplanned births and a generalized HIV epidemic [29-32]. Several large epidemiological studies have suggested an increased risk of HIV acquisition in women using high dose long-acting progestogens (the so-called ‘injectables’) [33], which means that these may have to be replaced with other long-acting hormonal methods such as CVRs, provided that they do not pose an HIV acquisition risk by causing inflammation.

The CVR that will be used in this trial is commercialized under the trade name of NuvaRing®, approved since 2001 by most countries within the European Union, and in the United Stated by the FDA. To date NuvaRing® is available in 32 countries and used by approximately 1.5 million women. NuvaRing® contains etonogestrel/ethinyl estradiol and is manufactured by N.V. Organon (a subsidiary of Merck & Co., Inc.,), Oss, the Netherlands.

We describe here the study protocol as it was initially designed. At time of submission of this paper, the study has started and data collection and analyses are ongoing.

### Study objectives

The study will be a multidisciplinary research project with two aims. The first aim will be to determine the safety of a CVR in women, with an emphasis on its effect on the vaginal microenvironment after different durations of use: the vaginal microbiome, biofilm formation on epithelial cells and rings, inflammation and immune activation in the vagina; and the second aim to investigate the feasibility, acceptability and adherence to vaginal ring use in Rwandan women, including attitudes towards a future multi-purpose vaginal ring for prevention of both pregnancy and STIs.

The study objectives are described in Table 1.

**Table 1:** **Study objectives**

	**Clinical trial**	**Social science component**
**Primary objective**	To assess the impact on the vaginal microbiome of the use of a CVR intermittently (3 weeks followed by 1 week off) or continuously.	To assess the level of acceptability and reported adherence to intermittent and continuous CVR use in women in Rwanda
**Secondary objectives**	To assess the general safety of the CVR.	To identify and describe the context specific attitudes and beliefs regarding family, family planning and gendered norms.
	To assess the vaginal biofilm formation according to ring use regimen. To determine the presence or absence of a biofilm on the CVRs after intermittent or continuous use by visualization of the biofilm. To assess the impact of CVR on vaginal inflammation and immune activation according to ring use regimen.	
**Exploratory objectives**		To explore the women’s beliefs and expectations regarding future potential use of a multi-purpose ring (contraception and HIV prevention). To explore how women and men in Rwanda perceive and experience risk related to unwanted pregnancy and HIV.

## Methods and design

### An integrated study design

The clinical trial will combine a clinical safety evaluation of the CVR with social science research on acceptability and adherence of ring use in Rwandan women.

**Figure 1:**
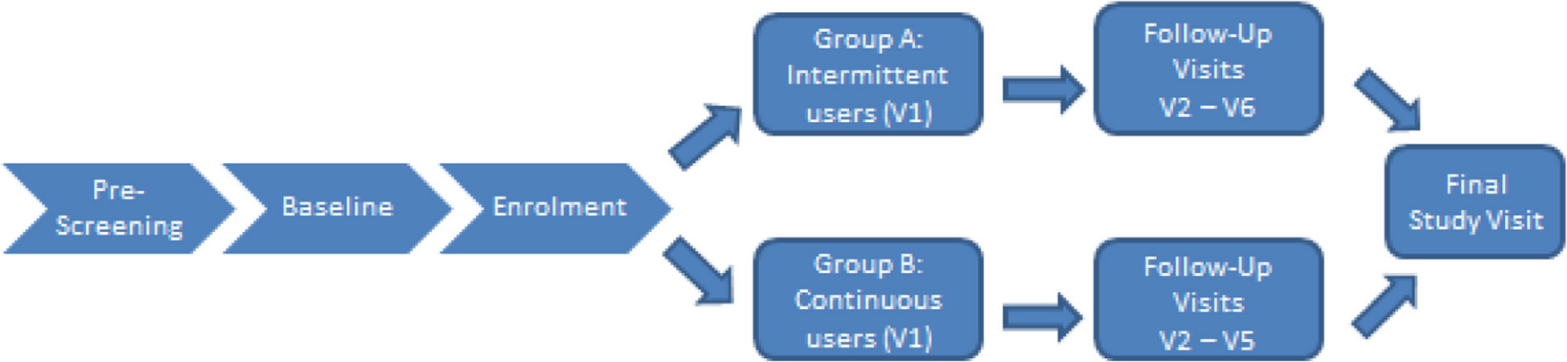
**Schedule of the clinical study.**

This will be an open label single-centre cohort study with the NuvaRing®. A total of 120 HIV-negative women will be randomized to an intermittent or a continuous regimen of ring use and will be followed for maximum 14 weeks, to determine general safety of the ring in the African context, and to determine differences in the vaginal microbiome and vaginal inflammation before and after use of a CVR (Figure 1). Qualitative research for the social science component will be performed to address the acceptability and adherence of intermittent or continuous CVR use in more depth.

### Study participants

**Figure 2:**
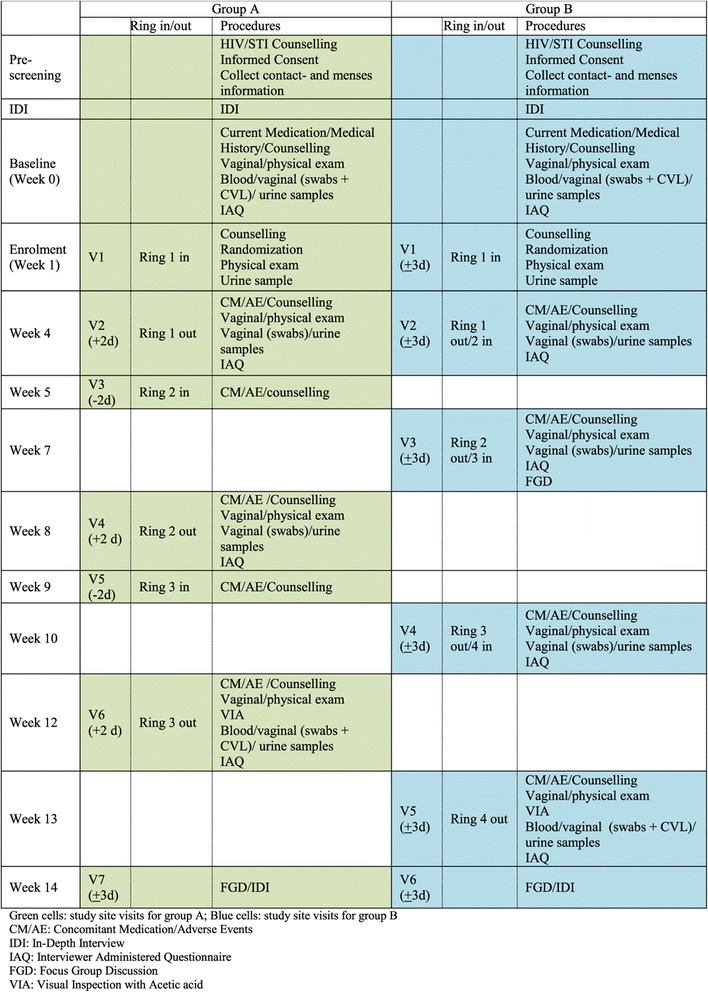
**Schedule of assessments.**

HIV negative, healthy women not using a modern contraceptive method (not including barrier methods) and who are interested in initiating hormonal contraceptive use, will be recruited and followed up at the Rinda Ubuzima (RU) research clinic and laboratory in Kigali, Rwanda. They will receive extensive counselling on all contraceptive methods available in Rwanda and on prevention of HIV and other STIs. They will be offered HIV and STI testing and condoms free of charge. The social science component of the acceptability study will include in-depth interviews (IDIs) and focus group discussions (FDGs) which will take place alongside the clinical study (Figure 2).

Please refer to Table 2 for the in- and exclusion criteria.

**Table 2:** **In**- **and exclusion criteria**

**Inclusion criteria**	» Able and willing to give informed consent/assent, according to national guidelines
	» Female who self-reports to be sexually active (meaning at least one penetrative vaginal coital act per month for the last 3 months prior to screening)
	» Between 18 to 35 years old, inclusive
	» Currently in good physical and mental health
	» Interested in initiating hormonal contraception
	» Able and willing to participate in the study as required by the protocol, this includes willing to undergo HIV testing and use a NuvaRing®
	» HIV negative at screening as confirmed by rapid HIV testing
**Exclusion criteria**	» Currently using a modern contraceptive method other than barrier methods
	» Use of a hormonal contraceptive method in the three months prior to the screening visit
	» Currently using antimicrobial medication
	» Pregnant on urine pregnancy test
	» History of cardiovascular disease
	» History of hysterectomy or genital tract surgery (including cervical polypectomy, dilatation and curettage, hysteroscopy, or laparoscopy) in the three months prior to the screening visit
	» History of complications with hormonal contraception or with contra indications for the use of hormonal contraceptives such as:
	o History or known predisposition for venous thrombosis
	o History of migraine with focal neurological symptoms
	o Diabetes mellitus with vascular involvement
	o History of pancreatitis or severe hepatic disease
	o Known or suspected hypersensitivity to any of the excipients of NuvaRing®
	» History of significant urogenital or uterine prolapse, undiagnosed vaginal bleeding, incontinence or urge incontinence, diagnosed chronic and/or recurrent vulvovaginal candidiasis, urethral obstruction
	» Participating in other clinical studies involving investigational products
	» Currently breastfeeding
	» Currently a smoker

### Sample size and power calculation

A total of 120 women will be enrolled in the clinical trial: 60 will be randomized to the intermittent regimen of ring use and 60 to the continuous regimen. This clinical study sample size calculation is based on the primary objective to assess the pre-post changes in the vaginal microbiome.

We require 95% power to detect clinically important changes in bacterial counts. Analyses of our on-going biomarkers study showed a difference of (−)1 log in overall *Lactobacillus* sp. counts and of (+)3 log in *G. vaginalis* counts between participants with and without BV as assessed by Nugent score, and standard deviations of 1 and 3 for changes in overall *Lactobacillus* sp. counts and *G. vaginalis* counts, respectively [34]. We have defined a change in bacterial count of 50% of the observed differences between women with and without BV as clinically important. Given this, we require 52 women in each study group. To correct for early withdrawals and women lost-to-follow up, we will randomize 120 women (60 in each study group) to ensure we have 104 women with primary endpoint data available.

For the qualitative research, sample sizes will be determined by when data saturation is reached.

After pre-screening, IDIs will be conducted. Midway through the project, FGDs (8–12 women in each group) will be conducted as well as at the end of the project. In addition, IDIs will be conducted with women and with male partners of participants.

### Randomization and follow-up

#### Informed consent process

All informed consent (IC) procedures (including assessment of understanding and literacy) will be conducted by qualified staff members identified by the principal investigator (PI), in the language chosen by the participants and according to the requirements described in the Helsinki Declaration, in Rwandan legislation and in the Good Clinical Practice (GCP) guidelines. Participant information sheets and consent forms in the local language will be provided to the study participants or legally authorized representatives and witnesses for their review. After the IC procedure, the participants and, in case they are of minor age (age below 21 and unmarried in Rwanda), the parents or guardians will be asked to confirm their willingness to participate in the study by signing (or thumb-printing whenever they are illiterate) the consent form. The RU standard operating procedure (SOP) for IC will be followed.

If a participant (or parent or guardian) is unable to read or write, an independent witness will take part in the IC discussion and his/her signature will be obtained. Participants will be informed that participation in the study is completely voluntary and that they can withdraw from the study at any time without any negative consequences.

Separate IC forms will be given to all study participants and partners who agree to participate in the FGDs and IDIs.

#### Clinical part

This will be an open-label study because the differences in ring use (intermittent or continuous use) cannot be blinded. Individual participants will be randomized and treatment allocation will be concealed until a participant has provided informed consent, is confirmed eligible, is included in the study and inserted the CVR.

After randomization the follow up visits will be planned to coincide with times of removal and insertion of the ring (Figure 2). Participants will be asked to complete Interviewer Administered Questionnaire (IAQ) including adherence related questions.

Every effort will be made to ensure participants adhere to this visit schedule and in such way that their confidentiality and privacy will be protected.

#### Social science part

Women participating in IDIs will be purposively selected according to age/parity and experience with contraception in order to have a wide variation of key characteristics.

A sub-sample of women will be purposively selected according to specific criteria i.e. vaginal practices, ring removal and/or expulsions, parity and experience with contraception to participate in FGDs.

After trial completion, women who will not yet have participated in any other FGDs will be selected based on ring removal and/or expulsions. Participants and partners will be invited to participate in end trial IDIs.

### Diagnostic tests and laboratory procedures

All treatable genital infections identified using the syndromic approach and any missed infections but identified later by laboratory testing will be treated according to RU SOPs based on national guidelines. Table 3 lists diagnostic tests that will be performed using validated test kits or methods, and procedures [34]. Table 4 lists the research tests that will be performed.

**Table 3:** **Diagnostic tests that will be performed in the study**

**Facility where the tests will be performed**	**Tests that will be performed**
**Laboratory at RU in Rwanda**	» Urine analysis for pregnancy using QuickVue hCG (Quidel);
	» HIV testing followed RU SOPs, which are based on the Rwandan national guidelines, using DetermineTM
	HIV1/2 (Abbott Diagnostic Division, Hoofddorp, The Netherlands) as first test and Uni-Gold HIV (Trinity, Berkeley Heights, New Jersey, USA) as second test when the first test is reactive;
	» Wet mount microscopy;
	» HSV-2 (HerpeSelect 2 ELISA (Kalon Diagnostics) and syphilis testing assessed with RPR (Spinreact Reactivos, Girona, Spain), followed by TPHA (Spinreact Reactivos, Girona, Spain)
**Laboratoire National de Référence**, **Kigali**	» CT/NG molecular testing using the Presto CT/NG kit of Goffin Molecular Technologies, The Netherlands;
	» Elisa for HIV (VIRONOSTIKA HIV Uni-Form II Ag/Ab, bioMérieux Marcy l'Etoile, Marcy l'Etoile, France) if the first 2 tests done at the laboratory of RU showed discordant results.

**Table 4:** **Research tests that will be performed in the study**

**Facility where the tests will be performed**	**Tests that will be performed**
**Laboratory at RU or in another laboratory in Rwanda**	1. A vaginal smear will be used for BV diagnosis and a microscopic description of the vaginal flora. The current gold standard for BV diagnosis in research settings is the Nugent score. This method relies on the semi-quantification of the bacterial morphotypes observed in a vaginal smear after Gram staining. The modified Ison & Hay grading is another system to describe the vaginal flora and provides more details on the Lactobacillus species compared to the Nugent score. The Nugent score will be used to diagnose BV and offer treatment to the women, whereas the modified Ison & Hay grading will be used for research purposes only.
	2. Luminex and ELISA cytokine testing will be used to identify women who had vaginal inflammation. All samples that will be showing elevated cytokine levels, as well as an equal number of controls not showing elevated cytokine levels, will be tested further for the presence of proteins involved in inflammatory pathways.
**HIV**/**STI reference laboratory at ITM**	1. Vaginal swabs will be tested with molecular amplification techniques for the characterization and quantification of the vaginal microbiome. Smears and an aliquot of the urine samples will be fixated and shipped to ITM to study the changes in phenotype of BV-related bacteria (from dispersed forms to adhesive forms on epithelial cells) to identify biofilm formation. Dispersed and adherent bacteria will be assessed in a multicolour analysis with a mix of differently stained group-specific and universal bacterial probes and visualized by confocal imaging.
	2. The biofilms formed on the CVRs will be visualized using crystal violet staining and/or abbreviation.

### Investigational product

NuvaRing® is manufactured by N.V. Organon (a subsidiary of Merck & Co., Inc.,), Oss, the Netherlands and contains 11.7 mg etonogestrel and 2.7 mg ethinylestradiol. The CVR releases etonogestrel and ethinylestradiol at an average amount of 0.120 mg and 0.015 mg, respectively per 24 hours, over a period of 3 weeks. The CVR is approved since 2001 by most countries within the European Union, and in the United States by the Food and Drug Administration.

### Safety

Participants will be asked to inform the study staff of any medical problems while they are taking part in the study. Adverse events (AEs) will be collected until the final ring removal visit and will also be identified during laboratory testing, medical histories and physical examinations. Treatment for AEs possibly/probably/definitely related to study participation will be provided by the study clinic at no cost to the participant. All clinical and laboratory toxicities will be managed according to standard medical guidelines. All AEs will be followed up until resolution.

Whenever a participant becomes pregnant during the study, she will be followed up by the study team until delivery, and the pregnancy outcome will be collected and documented. Any pregnancy outcome that meets any criteria for Serious Adverse Event (SAE) reporting (e.g. congenital anomalies) will be reported as SAE to the concerned bodies and included in the study report.

All SAEs whether or not deemed drug related or expected will be reported within 24 hours (one working day) to the pharmacovigilance of the clinical trials unit (CTU) of ITM.

Line listings of all reported SAEs will be sent to the Institutional Review Board (IRB) of the ITM and the Ethics Committee (EC) of the University Hospital of Antwerp (UZA) annually and to the Rwanda National EC (RNEC) case by case. RU will also submit annual line listings of all AEs to the (RNEC).

An independent data safety monitor (gynecologist) will review the SAE reports with special attention to cardiovascular events (for example venous thrombosis) which may be associated with the use of hormonal contraception.

### Monitoring and quality assurance

This study will be monitored by ITM in accordance with regulations applicable to clinical trials, including GCP and GCLP (Good Clinical Laboratory Practices) requirements, and sponsor-specific SOPs. The social science component research will adhere to the guidelines established by the American Anthropological Association.

A quality system has already been put in place at RU by the ITM in previous collaborative projects and ITM will ensure that all laboratory activities including specimen transport, processing, testing, result reporting and storage were conducted in accordance with clinical trial quality requirements. The GCLP guidelines will be followed and the laboratory will perform testing according to the SOPs which will be documented in the analytical plan.

### Data management

Due to the integration of quantitative and qualitative methods in this study four different types of databases will be set up (Table 5).

**Table 5:** **Different types of databases that will be used in the study**

**Types of database**	**Characteristics**
**Clinical trial database**	will be programmed, tested and validated prior to study start and for every modification during the study if applicable
**Quantitative behavioural**/**adherence**/**acceptability database**	will be tested prior to use but will not be fully validated according to a clinical trial validation protocol. Revisions to this database after study start could be necessary when the initial qualitative research show that our questionnaires are not optimal.
**Laboratory databases**	will be developed for the laboratory tests done in batches. Data in these databases will be entered and double-checked manually by qualified laboratory professionals according to the study SOP. The data may be combined with variables from the above two databases for analysis.
**Qualitative behavioural**/**adherence**/**acceptability data**	IDI/FGD transcripts and coding

The clinical trial electronic case report form (eCRF) and database will be designed by the data manager of the CTU (ITM) who will also perform the data management of the clinical trial in collaboration with the assigned site staff (country coordinating investigator, PI, data entry clerk).

Private information on trial participants will be handled confidentially. A specific study code and initials will be used on the trial source document template, the eCRF and on any other paper documents or electronic files.

Access to all paper documents and electronic files will be restricted to authorized study staff. The study computers and eCRFs will only be accessible via a login with personal username and password.

Data registered on the source document template will be recorded via eCRFs into the clinical trial database, using OpenClinica. OpenClinica is the world's leading open source clinical trials software for electronic data capture and clinical data management. OpenClinica supports GCP quality standards, guidelines and requirements such as the Code of Federal Regulations Title 21 part 11 (e.g. electronic signature, electronic audit trail). The experience in the use of an open source data management system will strengthen the capacity of the local site.

### Data analysis

#### Clinical analysis

The statistical analysis of the primary objective and secondary objective one of the clinical trial will be performed by the study statistician at the CTU (ITM) according to a Statistical Analysis Plan.

The objectives (Table 1) will be reached by describing and comparing the variables of interest in women randomized to either intermittent vaginal ring use (group A) over a 12 week study period or continuous ring use (group B) over a 13 week study period.

In general, analyses will be performed using an "as-randomized" all-participants-treated approach. All women randomized and who insert the CVR at the enrollment visit will be included in the analysis under the group they are randomized to, even if they discontinue before visit 2 (week 4). For the analyses requiring vaginal or urine samples, all women with available data will be used, i.e. women without a sample prior to or during CVR use will be excluded.

Participants in each treatment group will be described with respect to baseline characteristics. The primary analysis is based on the quantitative real time polymerase chain reaction (qPCR) vaginal flora data. This data will comprise different species and will be recorded at baseline (week 0), and at 3 (group A) or 4 (group B) time points during CVR use.

The presence/absence of each species during CVR use will be analyzed using repeated measures logistic regression models, with random intercepts and random slopes over time for each woman and fixed effects for randomization group (intercept and slope).

The incidence of signs and symptoms, study/ring use withdrawals, adverse experiences and STIs will be compared among randomization groups over the full study period using Fisher's exact test. Changes in Nugent scores and Ison & Hay grading of the vaginal flora will be graphically depicted and analysed using repeated measures (logistic) regression models.

The statistical analysis of secondary objectives 2 and 3 are research and data driven, no prior statistical analysis plan will be made but the statistical analysis methods will be fully described in the resulting reports and/or publications. Presence/absence of dispersed/adherent form of BV-related bacteria and of biofilms will be analyzed using repeated measures logistic regression models.

#### Qualitative analysis

All IDIs and FGDs will be audio-recorded and will be followed by a debriefing session. After transcription and translation, data will be uploaded into Nvivo 9.

For the primary objective, the IDI and FGD data will be analysed through a deductive, content-analytical approach to assess if the components of the theoretical framework developed by Woodsong [9] are valid in the given setting. New elements that emerge from the data will be integrated into the framework. Selected statistical analysis will be conducted on the data of the individual asked questionnaires, the ballot box questionnaires and the self-rating adherence scales. This quantitative analysis will be largely descriptive.

To analyse the IDIs and FGDs for the secondary objectives, open coding methodology based on a data driven framework will be developed by the research team.

A descriptive analysis of the data from the individual asked questionnaires will be conducted to address the exploratory objectives, in parallel with coding of the IDIs and FGDs based on a risk perception attitude framework supplemented by the research team as themes emerge.

All coding and analysis will be performed by at least two qualitative researchers to compare their coding and reach consensus on convergent issues.

### Ethics

The protocol and all study documents were reviewed and approved by the IRB of the ITM, the EC of UZA and the RNEC, the National Health Research Committee and to the Rwandan Ministry of Health (MoH). Approval for importing vaginal rings was obtained from the Rwandan MoH. No study activities will be performed before approval from all these bodies is obtained.

Yearly re-approval from the RNEC will be sought after submission of the annual report.

The study will be carried out according to the principles stated in the Declaration of Helsinki, all applicable national and international regulations and International Conference on Harmonization and World Health Organization GCP guidelines.

### Steering committee and trial management group

A Trial Management Group (TMG) will be in charge of the day-to-day management of the clinical trial.

A Steering Committee (SC) will be set up to manage and supervise the entire project. The SC will consist of the TMG and at least one study member of each partner institution.

### Archiving

The PI and the sponsor must maintain adequate and accurate records to enable the conduct of the study and the verification of the data.

After completion of the study, the site investigator file and the sponsor trial master file will remain available for internal audits and/or inspections of regulatory authorities for a period of twenty years in accordance with the Belgian legislation.

## Discussion

Before implementing an intervention, it is beneficial to investigate whether this intervention, if proven effective, will be accepted and used by the local population. Therefore a mixed method approach was chosen for our study. However including a sociological component to a clinical study can be challenging as sexual behavior questioning in research trials is not straight forward due to several factors, for example the public opinion or the risk of being excluded from the study, and participants may give inaccurate reports. For this reason different sources such as the questionnaires, diary cards and data obtained from the IDIs and FGDs will be used to triangulate and assess the validity of the data.

Another challenge is to follow the protocol as designed at the start of a clinical trial. As it is an intricate task to predict every need that will arise during the course of the study, it could be that, after a cautious assessment of the pros and cons, changes are required during the study. For our study, two main changes occurred: an additional cervical swab taken at baseline for the rapid detection of CT using the BioChekSwab (a rapid self-contained, two step enzyme detection system) and the range of male partners that were invited for an IDI was broaden from partners from women who participated in the end trial IDI or FGD to all partners. These amendments to the protocol were submitted and approved by all involved review bodies.

Multi-purpose rings require continuous use. However as the CVR used in our study, the NuvaRing®, releases the contraceptive hormones etonogestrel and ethinylestradiol over a period of 3 weeks (with a maximum of 4 weeks after which the contraceptive effect cannot be guaranteed anymore), it was not possible to implement continuous use. Our continuous use study regimen, i.e. replacing the ring every 3 weeks, approaches the continuous use as close as possible.

Due to the expected short recruitment period and the use of commercially available and approved CVRs, no formal data safety monitoring board (DSMB) will be set up and an independent data safety monitor will be appointed. In case of major safety concerns, this person will request the sponsor to halt recruitment of the trial and/or to organize a formal DSMB with a complete overview of all available safety data.

The proposed study will prepare the study community in Rwanda for vaginal ring use. It will improve the study instruments (particularly related to adherence and acceptability) of the RU research centre and will strengthen their research capacity even further. In a wider context, the project results will be shared within our extensive network of microbicide researchers and in particular within the network of the single- and multipurpose vaginal ring researchers. Our findings will thus inform the development of future multipurpose vaginal rings for Africa and future vaginal ring trial design. Finally this study may lead to the introduction of contraceptive vaginal rings on African markets.

### Study status

The study is ongoing. Data collection and data analysis are ongoing.
